# Efficacy of High Intensity Exercise on Disease Activity and Cardiovascular Risk in Active Axial Spondyloarthritis: A Randomized Controlled Pilot Study

**DOI:** 10.1371/journal.pone.0108688

**Published:** 2014-09-30

**Authors:** Silje Halvorsen Sveaas, Inger Jorid Berg, Sella Aarrestad Provan, Anne Grete Semb, Kåre Birger Hagen, Nina Vøllestad, Camilla Fongen, Inge C. Olsen, Annika Michelsen, Thor Ueland, Pål Aukrust, Tore K. Kvien, Hanne Dagfinrud

**Affiliations:** 1 National Advisory Unit on Rehabilitation in Rheumatology, Department of Rheumatology, Diakonhjemmet Hospital, Oslo, Norway; 2 Department of Health Sciences, University of Oslo, Oslo, Norway; 3 Department of Rheumatology, Diakonhjemmet Hospital, Oslo, Norway; 4 Research Institute of Internal Medicine, Oslo University Hospital, Rikshospitalet, Oslo, Norway; 5 Section of Clinical Immunology and Infectious Diseases, Oslo University Hospital, Rikshospitalet, Oslo, Norway; 6 Institute of Clinical Medicine, University of Oslo, Oslo, Norway; 7 K.G. Jebsen Inflammatory Research Center, University of Oslo, Oslo, Norway; University of Texas Health Science Center at Houston, United States of America

## Abstract

**Background:**

Physical therapy is recommended for the management of axial spondyloarthritis (axSpA) and flexibility exercises have traditionally been the main focus. Cardiovascular (CV) diseases are considered as a major health concern in axSpA and there is strong evidence that endurance and strength exercise protects against CV diseases. Therefore, the aim of this study was to investigate the efficacy of high intensity endurance and strength exercise on disease activity and CV health in patients with active axSpA.

**Methods:**

In a single blinded randomized controlled pilot study the exercise group (EG) performed 12 weeks of endurance and strength exercise while the control group (CG) received treatment as usual. The primary outcome was the Ankylosing Spondylitis (AS) Disease Activity Score (ASDAS). Secondary outcomes included patient reported disease activity (Bath AS Disease Activity Index [BASDAI]), physical function (Bath AS Functional Index [BASFI]), and CV risk factors measured by arterial stiffness (Augmentation Index [Alx]) and Pulse Wave Velocity [PWV]), cardiorespiratory fitness (VO_2_ peak) and body composition. ANCOVA on the post intervention values with baseline values as covariates was used to assess group differences, and Mann Whitney U-test was used for outcomes with skewed residuals.

**Results:**

Twenty-eight patients were included and 24 (EG, n = 10, CG, n = 14) completed the study. A mean treatment effect of −0.7 (95%CI: −1.4, 0.1) was seen in ASDAS score. Treatment effects were also observed in secondary outcomes (mean group difference [95%CI]): BASDAI: −2.0 (−3.6, −0.4), BASFI: −1.4 (−2.6, −0.3), arterial stiffness (estimated median group differences [95% CI]): AIx (%): −5.3 (−11.0, −0.5), and for PVW (m/s): −0.3 (−0.7, 0.0), VO_2_ peak (ml/kg/min) (mean group difference [95%CI]: 3.7 (2.1, 5.2) and trunk fat (%): −1.8 (−3.0, −0.6). No adverse events occurred.

**Conclusion:**

High intensity exercise improved disease activity and reduced CV risk factors in patients with active axSpA. These effects will be further explored in a larger trial.

**Trial Registration:**

ClinicalTrials.gov NCT01436942

## Introduction

Axial spondyloarthritis (axSpA) including ankylosing spondylitis (AS) is characterized by inflammatory back pain and reduced spinal mobility [1]. Over the past decades, the management of many rheumatic diseases has been revolutionized by improvements in diagnostic techniques and medication leading to a more active treatment approach for patients with axSpA [2]. Physical therapy with supervised exercise is a cornerstone in the treatment of axSpA together with anti-inflammatory medications such as non-steroidal anti-inflammatory drugs (NSAIDs) and tumor necrosis factor (TNF)-inhibitors [2]. There are no definite recommendations on type of exercise and intensity, and traditionally flexibility exercises at a low intensity level have been recommended [3].

A Cochrane review concluded that physical therapy interventions, mainly consisting of flexibility exercises, have beneficial effects on pain, spinal mobility, physical function and patient global assessment in AS patients, but the effect sizes are small [4]. Additional support for exercise as a part of the treatment repertoire in axSpA is the evidence of increased risk of cardiovascular diseases (CVD) in these patients [5]–[7]. For healthy adults, cardiorespiratory exercise is recommended in guidelines for CVD prevention [8], and high intensity endurance exercise has been shown to be more effective than low intensity endurance exercise [9]. In addition, the health benefits of strength exercise in CVD prevention are well established [10]. However, the evidence for effects of high intensity endurance and strength exercise on disease activity and cardiovascular (CV) -risk is limited in patients with axSpA.

Therefore, the aim of this study was to investigate the efficacy of high intensive endurance and strength exercise on disease activity and CV-risk in patients with active axSpA.

## Methods

The protocol for this trial and supporting CONSORT checklist are available as supporting information; se Checklist S1 and Protocol S1.

### Design

This study was a single blinded randomized controlled pilot study comparing an exercise group (EG) with a treatment as usual control group (CG). The intervention lasted for 12 weeks, and participants were examined at baseline and post intervention. All the participants provided written informed consent to participate, and all procedures followed the Helsinki declaration. The study was approved by the Regional Committee for medical and health research Ethics of South East Norway (reference: 2011/1468) and is listed in ClinicalTrials.gov (NCT01436942).

### Participants

Patients fulfilling the following criteria were considered eligible: axSpA according to the Assessment of SpA International Society (ASAS) classification criteria [11], age 18–70 years, no change in TNF-inhibitor use during the last 3 months, moderate to high disease activity (Bath AS Disease Activity Index [BASDAI] ≥3.5) and not performed regular endurance or strength exercise during the last year (>1 hour per week). Exclusion criteria were established CVD, other co-morbidity involving reduced exercise capacity, inability to participate in weekly exercise sessions in Oslo and pregnancy. The study was carried out at Diakonhjemmet Hospital in Oslo between October 2011 and June 2012, with recruitment of patients during the first six months. No formal sample size consideration was performed for this intervention due to the exploratory design of the study.

### Intervention

The exercise program followed the American college of sports medicine (ACSM) recommendations for maintenance and improvement of cardiorespiratory- and muscular fitness [10]. Patients in the EG were encouraged to exercise 40–60 minutes three times a week for twelve weeks. Twice a week the exercise sessions were carried out at a fitness center with individual supervision from a physical therapist (SHS). These sessions consisted of both endurance and strength training. The endurance training was high intensity interval training on a treadmill for 40 minutes (four minutes walking/running at 90–95% of maximal heart rate [HR] followed by three minutes of active resting at 70% of maximal HR repeated four times) [12]. The strength training was 20 minutes with external load for major muscle groups (individually adapted, six exercises, eight to ten repetitions maximum, two to three sets). The strength program consisted of the following exercises: bench press with weight manuals or seated in a chest press machine, squat with weight or leg press machine, rowing exercise with weight manuals, exercises for triceps and biceps in a fitness machine and an abdominal stabilization exercise (abdominal bridge).

Once a week the participants exercised individually minimum 40 minutes of endurance training, either an additional session with interval training or a session with long-distance training (above 70% of maximal HR). The exercise program was individually adapted to the participant's fitness level and a physical therapist (SHS) was responsible for an adequate progression of the intensity level during the exercise period.

The maximal HR of each participant was determined during the baseline test and the HR was controlled with a HR monitor to individually tailor the intensity. Attendance at the supervised exercise sessions was recorded by the physical therapist (SHS). The weekly self-imposed exercise session was first recorded in the pulse watch and thereafter weekly reported to the physical therapist. To fulfill the exercise protocol the participants had to attend at least 80% of the planned exercise sessions.

Participants in the CG were asked to not start exercising during the intervention period. The CG was introduced to the exercise program post-intervention to reduce drop out.

### Procedures of assessments

Assessments for efficacy and safety were performed at baseline and after 12 weeks (end of study) and included questionnaires, clinical examinations and laboratory measurements.

Personal characteristics, comorbidities and medication were self-reported in a questionnaire. All physical (spinal mobility, body weight/height, waist circumference and body composition) and performance (VO_2_ peak) based measures were recorded by an experienced physical therapist (CF). The assessments of blood pressure (BP), HR and arterial stiffness were performed by an experienced rheumatologist (IJB).

### Primary outcome measure

The primary outcome was disease activity assessed by the AS Disease Activity Score (ASDAS) [13]. ASDAS is reported to be a valid measure of disease activity as it has shown acceptable concurrent validity with both patients and physicians global assessments. ASDAS is a composite continuous score consisting of three patient reported items in addition to C-reactive protein (CRP).

### Secondary outcomes measures

#### Disease specific measures

Disease activity was also measured by the patient reported index BASDAI [14]. The BASDAI consists of six items related to major symptoms in AS (fatigue, spinal pain, joint pain, tenderness and degree and length of morning stiffness). BASDAI is reported to be valid as it reflects the entire spectrum of the disease and is sensitive to changes over time [14]. Physical function was measured with Bath AS Functional Index (BASFI) which is a disease specific index that consists of eight questions regarding physical functioning and two questions reflecting the patient's ability to cope with everyday life [15]. For both BASDAI and BASFI each question was answered on a 10 point numeric rating scale, and a sum score was calculated (0–10, 10 =  worst). Spinal mobility was measured by Bath AS Metrology Index (BASMI) [16]. BASMI includes five objective measurements of spinal mobility, and the score is 0–10 for each component, and the mean of the five scores produced a BASMI score from 0–10 (10 =  worst).

#### Cardiorespiratory fitness

Cardiorespiratory fitness was assessed with a maximal walking treadmill test for estimation of peak oxygen uptake (VO_2_ peak) as previously described [17]. Resting HR was measured after at least five minutes rest in a supine position and several recordings were made until two measurements differed by ≤5 heart beats, and the mean was then calculated.

#### Arterial stiffness

We measured arterial stiffness after at least five minutes of rest in a supine position, using the Sphygmocor apparatus (AtCor, Sidney, Australia). Several recordings were made from each patient and only those of high quality, according to standardized criteria [18], were chosen for further analyses. Augmentation Index (AIx) was estimated through pulse wave recordings at the radial artery. The pulse waves of the central arteries were derived by applying a validated transfer factor [19]. For measurements of Pulse Wave Velocity (PWV), we recorded the pulse at the carotid artery and the femoral artery. The distance between these points was measured (subtracting the distance between carotid artery and sternal notch from distance between femoral artery and sternal notch). The time of wave travel was simultaneously recorded with electrocardiogram. PWV was calculated by dividing the distance with the time, expressed in m/s.

#### Body composition

Body weight and body height were measured with calculation of body mass index (BMI) (kg/m^2^). Waist circumference was measured with a measuring tape at the height of umbilicus with the patient lying in supine position. Dual-energy X-ray absorptiometry (DXA) was used to assess body composition (body fat) and DXA is reconized as a validated reference method for assessing body composition [20].

#### Blood pressure

Brachial BP was measured in a supine position after at least five minutes rest using Omron M7 (Kyoto, Japan). Several measurements were performed until two differed by ≤5 mmHg in both systolic and diastolic BP and the average of these two measurements were calculated.

#### Inflammatory markers, cytokines and lipids

Blood samples were drawn after at least four hours fasting, and at least 48 hours after the last exercise session, and then analyzed for CRP, total cholesterol (TC) and high density lipoprotein cholesterol (HDL-c) by COBAS 6000 (Roche Diagnostics, Basel, Switzerland). Low density lipoprotein cholesterol (LDL-c) was calculated from Freidewalds formula [21]. Erythrocyte sedimentation rate (ESR) was measured using the Westergren method. In addition, blood samples were collected in EDTA containing vacutainer tubes, plasma were isolated and frozen at −80°Celsius and batch analyzed by enzyme immunoassays (EIAs). The following were analyzed: interleukin (IL)-6, IL-23 (Abcam, Cambridge, UK), IL-17a (Peprotec, Rocky Hill, NJ, USA), IL-18 and soluble TNF receptor 1 (sTNF-R1) and 2 (sTNF-R2) (RnDsystems, Stillwater, MN) at the end of the study. The inter- and intra-assay coefficients of variation were less than 10% for all EIAs.

#### Safety

Safety was considered as absence of a flare up in disease activity and was defined in terms of stable or decreased self-reported disease activity (ASDAS and BASDAI) and acute phase reactants (CRP and ESR). Further, we included the report of any adverse events in the definition of safety.

### Randomization and blinding

Allocation to EG or CG followed a computer-generated randomization list prepared by a study-independent statistician. Block-randomization with a block-size of four was used. The group assignment was concealed in envelopes and revealed consecutively after baseline testing. The recruiting investigator was unaware of the next participant's allocation. The assessors were blinded for group assignment and participants were instructed not to reveal any information regarding their group attachment or treatment program to the assessors.

### Statistical analyses

Statistical analyses were performed using SPSS version 21. The main statistical analyses were performed on the per-protocol population since this was an explanatory study [22]. The per-protocol population consisted of all randomized patients following the exercise protocol (at least 80% exercise attendance in EG and no exercise in CG). We used analysis of covariance (ANCOVA) on the post-intervention values to assess the group differences with p-values, mean difference and 95% CI. Baseline values were included as covariates. We assessed the normality assumptions of the ANCOVA models by pp-plots of the residuals. The residuals for ESR, CRP, IL-6, AIx and PWV were not normally distributed, and group differences in change from baseline were analyzed using the Mann-Whitney U-test with median and corresponding 95% confidence limits by the Hodges-Lehman estimator. P-values <0.05 were considered to be statistically significant. We considered all analyses to be exploratory, and did not adjust for multiple testing.

## Results

### Participant flow

**Figure 1:**
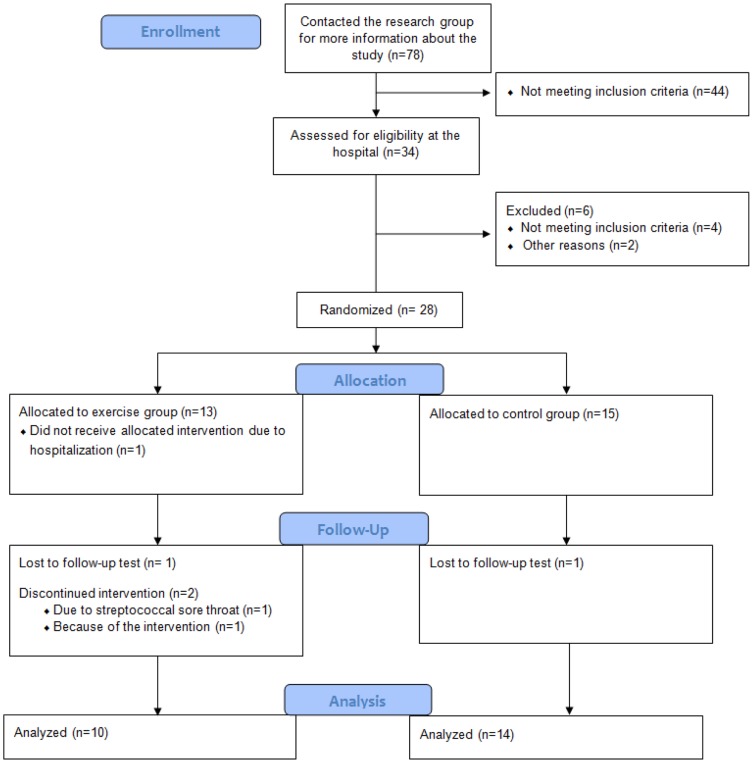
Flow of participants through the randomized controlled pilot study. EG: exercise group. CG: control group.

Thirty-four patients were assessed for eligibility (Figure 1). Six patients were excluded or withdrawn before randomization. Twenty-eight patients were randomized, 13 in EG, 15 in CG. One patient in the EG withdrew immediately after randomization due to hospitalization. Three patients dropped out during the intervention period (EG = 2, CG = 1). In the EG, one patient dropped out because of a streptococcal infection in the throat and one patient dropped out because the intervention was physically challenging and time consuming. In the CG, one patient did not perform testing after 12 weeks.

All the completing participants in the EG followed ≥80% of the exercise protocol, and reached their targeted HR during the endurance exercise sessions. In the CG, none of the completing participants reported that they had exercised during the intervention period, but one patient in the CG started with TNF-inhibitor four weeks after the baseline assessment. Following this, the per-protocol population consisted of 24 patients, 10 in the EG and 14 in the CG.

### Characteristics

There were significantly more males in the CG compared to the EG (p = 0.01), and the groups differed in weight (p = 0.01) and height (p = 0.01), presumable due to the gender difference (Table 1). Baseline characteristics were similar across the groups for other variables (Table 1).

**Table 1:** Baseline descriptive of all patients, exercise group and control group.

	All patients, n = 24	Exercise group, n = 10	Control group, n = 14
Age, years, mean (SD)	48.5 (12.0)	46.6 (13.6)	49.9 (11.1)
Gender, male, n (%)	12 (50)	2 (20)^a^	10 (71)^a^
Current smoking, n (%)	3 (13)	1 (10)	2 (14)
Height, m, mean (SD)	1.75 (0.07)	1.71 (0.06)^a^	1.78 (0.07)^a^
Weight, kg, mean (SD)	79.5 (15.7)	70.0 (12.3)^a^	86.3 (14.6)^a^
Disease duration, years, mean (SD)	24.9 (15.8)	19.2 (19.8)	28.6 (11.9)
ASDAS, mean (SD)	2.6 (0.6)	2.3 (0.6)	2.7 (0.8)
BASDAI, mean (SD)	5.3 (1.4)	5.3 (1.4)	5.3 (1.4)
CRP, median (range)	2 (1, 23)	1 (1, 9)	2 (1, 23)
ERS, median (range)	7(1, 41)	10 (2, 41)	6 (1, 24)
Diabetes, n (%)	1 (4)	1 (10)	0 (0)
Hypertension, n (%)	4 (17)	1 (10)	3 (21)
NSAIDs, n (%)	18 (75)	8 (80)	10 (71)
TNF-inhibitor, n (%)	7 (29)	1 (10)	6 (43)
Anti-hypertensives, n (%)	4 (17)	0 (0)	4 (29)
Statins, n (%)	5 (21)	2 (20)	3 (21)

ASDAS, Ankylosing Spondylitis Disease Activity Score; BASDAI, Bath Ankylosing Spondylitis Disease Activity Index (0–10, 10 =  worst); CRP, C-reactiv protein; ESR, erythrocyte sedimentation rate NSAIDs, non-steroidal anti-inflammatory drugs; TNF, tumor necrosis factor, SD; standard deviation.

a Statistically significant differences between groups. Analysed with bivariate test as appropriate.

### Efficacy on disease activity

Although not statistically significant, there was an improvement in the primary outcome ASDAS score in the EG compared to the CG (adjusted mean between group difference −0.66, 95%CI [−1.37, 0.05], p = 0.07) (Table 2), whereas patient reported disease activity, BASDAI, improved significantly (adjusted mean between group difference −2.0, 95%CI [−3.6, −0.4], p = 0.02).

**Table 2:** Effects of high intensity exercise on disease activity, inflammatory markers and cytokines.

	Exercise group, n = 10		Control group, n = 14		Estimated mean group difference (95% CI)^a^	p-value
	Baseline	3 months	Baseline	3 months		
**Disease activity**						
ASDAS, mean (SD)	2.3 (0.6)	1.8 (0.9)	2.7 (0.8)	2.6 (0.8)	−0.7 (−1.4, 0.1)^a^	0.07
BASDAI, mean (SD)	5.3 (1.4)	3.3 (2.0)	5.3 (1.3)	5.2 (2.0)	−2.0 (−3.6, −0.4)^a^	0.02
BASFI, mean (SD)	2.6 (2.2)	1.5 (1.5)	3.1 (1.6)	3.1 (1.4)	−1.4 (−2.6, −0.3)^a^	0.02
BASMI, mean (SD)	2.3 (1.5)	2.0 (1.6)	3.0 (1.8)	2.9 (1.8)	−0.3 (−0.9, 0.3)^a^	0.32
**Inflammatory markers**						
ESR (mm/h), median (range)	10 (2, 41)	9 (5, 26)	6 (1, 24)	7 (1, 46)	−1 (−6, 2)^b^	0.40^c^
CRP (mg/L), median (range)	1 (1,9)	1 (1,12)	2 (1, 23)	3 (1, 13)	0 (−1, 2)^b^	0.89^c^
**Cytokines**						
IL-6 (pg/mL), median (range)	0.3 (0.2, 5.0)	0.3 (0.2, 16.2)	0.4 (0.2, 2.8)	0.3 (0.2, 2.4)	0.0 (−0.3, 0.5)^b^	0.95^c^
IL-17a (pg/mL), mean (SD)	82 (53)	72 (27)	103 (90)	105 (80)	−16 (−34, 2)^a^	0.08
IL-18 (pg/mL), mean (SD)	95 (20)	86 (15)	97 (37)	101 (43)	−13 (−30, 4)^a^	0.12
IL-23 (pg/ml), mean (SD)	123 (39)	103 (25)	95 (47)	113 (39)	−24 (−49, 1)^a^	0.06
sTNFr1 (ng/mL), mean (SD)	0.87 (0.23)	0.87 (0.16)	0.98 (0.20)	1.03 (0.31)	−0.05 (−0.18, 0.09)^a^	0.46
sTNFr2 (ng/mL), mean (SD)	3.0 (2.5)	3.0 (2.5)	3.9 (3.3)	4.0 (3.3)	−0.1 (−0.3, 0.2)^a^	0.64

Differences between the groups in post intervention (3 months) values, analyzed with ANCOVA with baseline values as covariates.

All BAS-instruments 0–10, 10 =  worst.

ASDAS, Ankylosing Spondylitis Disease Activity Score; BASDAI, Bath Ankylosing Spondylitis Disease Activity Index; BASFI, Bath Ankylosing Spondylitis Functional Index; BASMI, Bath Ankylosing Spondylitis Metrology Index; CRP, C-reactive protein; ESR, erythrocyte sedimentation rate; IL, interleukin; sTNFR, soluble tumor necrosis factor receptor.

a Estimated regression coefficients,

b Hodges-Lehman median estimator,

c Mann-Whitney U-test.

### Efficacy on cardiovascular risk factors

**Figure 2:**
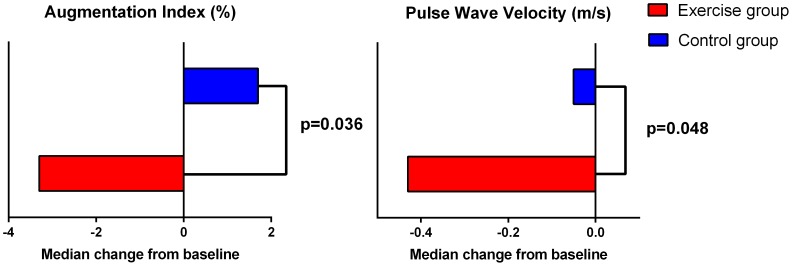
Median change in arterial stiffness after 3 months intervention of high intensity exercise, compairsons between control group and exercise group using Mann-Whitney U-test.

Arterial stiffness was significantly reduced in the EG compared to CG. The median changes in Alx and PWV for the EG and CG are presented in Figure 2. All changes were in favor of the EG. Further, significant treatment effects were seen for VO_2_ peak, resting HR, total body fat and abdominal fat (Table 3). A significant reduction in waist circumference was observed for subjects who had an increased waist circumference at baseline (males ≥94 cm and females ≥80 cm, analyzed: EG n = 6 and CG n = 12) in the EG compared to CG.

**Table 3:** Effects of high intensity exercise on cardiovascular risk.

	Exercise group, n = 10		Control group, n = 14		Estimated mean group difference (95% CI)^a^	p-value
	baseline	3 month	baseline	3 month		
**Cardiorespiratory fitness**						
VO_2_ peak (ml/kg/min) mean (SD)	36.0 (5.1)	39.4 (4.5)	36.8 (4.9)	36.3 (3.8)	3.7 (2.1, 5.2)^a^	<0.001
Resting HR, mean (SD)	64 (7)	59 (6)	59 (8)	62 (9)	−6 (−11, −1)^a^	0.02
**Body composition**						
Body weight total (kg), mean (SD)	70.0 (12.3)	70.6 (12.5)	86.3 (14.6)	86.8 (15.3)	0.5 (−1.1, 2.1)^a^	0.49
BMI (kg/m^2^), mean (SD)	24.0 (3.8)	24.2 (3.8)	27.1 (3.9)	27.2 (4.0)	0.0 (−0.6, 0.6)^a^	0.89
Waist circumference (cm)^2^, mean (SD)	94.7 (7.9)^b^	91.2 (6.8)^b^	101.3 (9.8)^b^	101.3 (10.1)^b^	−3.8 (−6.9, −0.8)^a^^,^ b	0.02^b^
Fat, total (%), mean (SD)	32.2 (7.4)	30.8 (7.2)	29.9 (6.2)	29.8 (6.2)	−1.4 (−2.1, −0.6)^a^	<0.001
Fat, abdominal (%), mean (SD)	29.8 (8.3)	27.5 (8.2)	29.6 (7.2)	29.2 (7.1)	−1.8 (−3.0, −0.6)^a^	<0.001
**Lipids**						
TC (mmol/L), mean (SD)	5.7 (1.2)	5.4 (1.2)	5.7 (1.0)	5.6 (0.7)	−0.2 (−0.8, 0.4)^a^	0.60
LDL-c, (mmol/L), mean (SD)	3.1 (1.3)	2.8 (0.9)	3.6 (0.9)	3.6 (0.9)	−0.5 (−1.1, 0.1)^a^	0.10
HDL-c, (mmol/L), mean (SD)	2.1 (0.3)	2.0 (0.5)	1.3 (0.4)	1.3 (0.5)	−0.3 (−0.7, 0.1)^a^	0.11
TC/HDL-c, (mmol/L), mean (SD)	2.8 (0.9)	2.9 (1.1)	4.9 (1.7)	4.6 (1.5)	−0.1 (−0.8, 0.7)^a^	0.88
**Blood pressure**						
Brachial SBP (mmHg), mean (SD)	126 (17)	126 (24)	130 (13)	124 (9)	7 (−3, 16)^a^	0.15
Brachial DBP (mmHg), mean (SD)	80 (8)	77 (11)	81 (7)	79 (8)	−1 (−8, 6)^a^	0.68
**Arterial stiffness**					**Estimated median group difference (95%CI)^c^**	
AIx (%), median (range)	21.5 (−8.7, 34.5)	18.5 (−17, 34.5)	16.4 (−5.0, 29,5)	15.5 (5, 30)	−5.3 (−11.0, −0.5)^c^	0.04^d^
PWV (m/s), median (range)	7.2 (5.8, 11.0)	6.2 (4.4, 10.4)	7.4 (5.3, 8.8)	6.6 (5.1, 8.1)	−0.3 (−0.7, 0.0)^c^	0.048^d^

Differences between the groups in post intervention (3 months) values, analyzed with ANCOVA with baseline values as covariates.

AIx, Augmentation Index; BMI, body mass index; DBP, diastolic blood pressure; HR, heart rate; HDL-c, high density lipoprotein cholesterol; LDL-c, low density lipoprotein cholesterol; PWV, Pulse Wave Velocity; SBP, systolic blood pressure; TC, total cholesterol; VO_2_ peak, peak oxygen uptake.

a Estimated regression coefficients;

b analyzing only subjects with an increased waist circumference at baseline (males ≥94 cm and females ≥80 cm, EG n = 6, CG n = 12),

c Hodges-Lehman median estimator,

d Mann-Whitney U-test.

### Efficacy on other secondary outcome measures

Significant treatment effects were seen for patient reported physical function, BASFI, but not for spinal mobility (BASMI) (Table 2). In general, there were no differences between the groups in change in inflammatory markers, although there was a trend towards a reduction in IL-17a and IL-23 in the EG compared to the CG (Table 2).

### Safety

Seven of 10 subjects in the EG had decreased disease activity, one had a stable disease activity and two increased their disease activity (+0.2 and +0.5 in ASDAS) from baseline to post intervention. There were no differences in change in inflammatory markers (CRP and ERS) between the groups. Furthermore, no adverse events were reported during the intervention, indicating that high intensity exercise was safe and well tolerated in patients with active axSpA.

## Discussion

This pilot study showed that high intensity endurance and strength exercise improved disease activity and reduced CV-risk factors in axSpA patients with active disease.

To our knowledge this is the first study aiming to examine whether patients with active axSpA could participate in a high intensity exercise program without a flare up in disease activity. Given the treatment effect of improved patient reported disease activity and stable inflammatory markers, the concept of high intensity exercise for axSpA-patients with active disease seems to be applicable. Our results are in accordance with a recently published case matched study by Stavropoulos-Kalinoglou et al. reporting that high intensity exercise significantly improved disease activity in patient with rheumatoid arthritis (RA) [23].

BASDAI has commonly been used as disease activity outcome measure in studies of effects of medication or exercise in patients with AS. Systematic reviews have reported treatment effects of TNF-inhibitors with effect size (ES) between 0.3 and 1.5 [24], and ES of exercise therapy (mainly flexibility exercises) to be between 0 and 0.8 in BASDAI [25]. In comparison, we found an ES of 1.4, suggesting that the intervention may have beneficial effects on patient reported disease symptoms. Thus, the improvement in BASDAI in our study supports that high intensity exercise may serve as an effective supplement to a pharmacological intervention.

Increased CV risk is an important factor for increased morbidity and mortality in patients with rheumatic diseases including those with axSpA. In the present study we found improvements in several CV risk factors. Central arterial stiffness measured as PWV is a validated marker of CV risk and has been used as a surrogate endpoint of CVD [26]. AIx is an estimation of central arterial pressure and has also been shown to predict CV mortality [27]. This is the first study to demonstrate improvements of arterial stiffness after exercise intervention in patients with axSpA, and in rheumatic diseases in general. However, improvement in arterial stiffness after an exercise intervention has been reported in young and middle aged healthy men [28], [29], and a cross-sectional study on RA patients reported that a higher level of self-reported physical activity was associated with lower arterial stiffness [30]. The magnitude of the reduction in arterial stiffness in the exercise group was AIx 3% and PWV 0.4 m/s, which is comparable to the reduction reported in studies on other populations [28], [29], [31]. A population study has shown that in young men, 10 years of vascular aging corresponds to an increase in PWV of 0.48 m/s and AIx of 9%, and in elderly men an increase in PWV of 1.36 m/s and AIx 1% [32]. Thus, the improvement in arterial stiffness in our study seems to be clinically meaningful in reducing CV risk. Arterial stiffness is determined by elastic properties of the artery wall (composition of elastin and collagen) and vasoconstrictor tone exerted by the smooth muscle cells [33]. The structural composition of elastin and collagen is believed to develop over years, and thus it seems unlikely that this would change after a short term intervention. Exercise can however alter arterial stiffness over a short period by modulation of the sympathetic-adrenergic tone either directly or through nitric oxide [29], [33], [34]. In addition, arterial stiffness can be affected by alterations of CV risk factors such as lipids, inflammation and especially BP [26]. In the present study these parameters showed no significant treatment effects. Our findings are supported by a study on healthy men, demonstrating favorable effects of exercise on arterial stiffness without changes in traditional CV risk factors, thus indicating a direct effect of exercise on the arterial stiffness [29].

In addition to arterial stiffness, the EG group improved VO_2_ peak and reduced resting HR, and hereby confirm that the exercise intervention was effective in improving cardiorespiratory fitness. In the exercise study by Stavropoulos-Kalinoglou et al., the RA patients showed similar improvements in VO_2_ peak [35]. Physical fitness is inversely related to CV risk in the general population [36]. Even small improvements in cardiorespiratory fitness are recognized as significant in reducing CV morbidity and mortality [37], and a prospective study reported that an increase in VO_2_ peak of 3.5 ml/kg/min corresponded to a substantial decrease in cardiac events in a broad heterogeneous cohort of middle-aged men [38]. We found a mean 3.7 ml/kg/min difference in change in VO_2_ peak in favor of the EG, thus indicating that the improvement in cardiorespiratory fitness seen in this study is of clinically importance in preventing CVD.

In addition, this study demonstrated significant treatment effects on total body fat and abdominal fat, although there were no differences in change in total body weight between the groups. Abdominal fat is associated with CV risk [39], and adipose tissue is described as an endocrine organ secreting inflammatory mediators like adipokines which play relevant roles in the pathophysiology of both CVD and inflammatory diseases [40]. Thus, the significant treatment effect on abdominal fat underline the potential of intensive exercise as a disease modifying treatment modality in patients with axSpA.

In contrast to these effects, and as mentioned above, we found no differences between the groups in change the plasma lipids and brachial BP. Two different meta-analyses concluded that exercise interventions have beneficial effects on BP, HDL-c and to some extend TC in the general population [41], [42]. Furthermore, Stavropoulos-Kalinoglou et al. found significant reductions in BP and improvement of lipid profile after three months and six months of exercise in RA patients [35]. In contrast and in line with our results, a recent RCT on a 12 week moderate intensity exercise intervention in AS did not show any significant reductions in TC [43]. The lack of effect on lipids and brachial BP in our study might be due to a small sample size, a short intervention period and that most patients were within the normal range of BP and lipids at baseline.

Enhanced inflammation is an important pathogenic mediator in various rheumatic disorders including axSpA. For healthy adults, an anti-inflammatory effect of exercise is reported [44], but to our knowledge, this has not been investigated in patients with axSpA. We found no significant effects of high intensity endurance and strengths exercise on plasma levels of a wide range of cytokines or cytokine modulators. However, the EG showed a trend towards a decrease in IL-17a and IL-23 as compared with the CG, and notably both these cytokines have recently been suggested to be major players in the pathogenesis of axSpA [45]. Interestingly, treatment with inhibitors of IL-17 [46] and IL-23/IL-12 [47] has recently shown promising results in reducing disease activity in patients with active AS. In order to obtain more insight in the effects of exercise on the inflammatory process in axSpA patients, exercise-response of relevant inflammatory markers should be further explored.

Our findings support a favourable effect of exercise on CV risk in an axSpA population. Whether these results may be extrapolated to other inflammatory diseases with increased CV risk, e.g. suchs as HIV infections [48], should be investigated.

A strength of the present study was the randomized controlled design. Further, the intervention was in accordance with the ASCMs exercise recommendations, was individualized and the patients were supervised in order to obtain the optimal dose of exercise as prescribed in the protocol. The intervention was individually adapted and the intensity was controlled, ensuring that the intended physiological goals were met. Thus, the results of this study may be considered as measures of efficacy, and the adherence to the program was confirmed by the physiological response in terms of increased cardiorespiratory fitness. The main limitation is that this was a small explanatory study carried out under close to optimal conditions. Further, the participants and the therapist were not blinded to treatment allocation, and lack of blinding is likely to exaggerate treatment effects on subjective outcomes [49]. The sample size is small, and negative results may not be true negative due to the insufficient power to show significant differences. Moreover, based on its explorative nature, a large number of parameters were compared in a relative small study population without correcting for multiple comparisons further underscoring that secondary outcomes should be interpreted with some caution.

## Conclusions

The results of the present study showed that high intensity cardiorespiratory and strength exercises improved disease activity and reduced CV risk factors in patients with active axSpA. The promising results of this pilot study will be further explored in a larger randomized controlled trial.

## Supporting Information

Checklist S1 **CONSORT Checklist.**
(DOC)

Protocol S1 **Trial Protocol.**
(DOC)
