# Supplementary material for: Efficacy of High Intensity Exercise on Disease Activity and Cardiovascular Risk in Active Axial Spondyloarthritis: A Randomized Controlled Pilot Study
Source: PLoS One. 2014 Sep 30;9(9):e108688. doi: 10.1371/journal.pone.0108688 (PMC4182541; doi:10.1371/journal.pone.0108688)
Supplement: Protocol S1 — Trial Protocol. (DOC) [file pone.0108688.s002.doc]

**Effects of exercise on disease activity and cardiovascular risk factors in patients with ankylosing spondylitis**

**SUMMARY**

**Background:** Exercise is recommended as a cornerstone in the treatment of ankylosing spondylitis (AS) together with medication. Last years, increased risk of cardiovascular diseases in patient with inflammatory diseases is reported, probably caused by inflammation and increased prevalence of traditional risk factors. In both healthy adults and other patient groups, cardiorespiratory and muscular strength exercises have been shown to have a positive effect on inflammation as well as on cardiovascular risk factors. To our knowledge this has not been shown in patients with AS.

**Objective:** The aim of this study is twofold: firstly, to investigate the effects of a supervised exercise program on disease activity and cardiovascular risk factors in patients with AS, and secondly, to examine acute effects on physiological responses to exercise.

**Design:** Single blind randomized controlled trail.

**Participants:** Patients who were classified with high disease activity in a cross sectional study carried out between 2008 and 2010 will be invited to participate (REK nr: s-03066 and S-02059). In addition, patients will be recruited from the rheumatologic outpatient clinic at Diakonhjemmet Hospital.

**Intervention:**  Supervised cardiorespiratory and muscular strength exercise program, one hour three times a week for twelve weeks.

**Primary outcome:** Disease activity (inflammatory markers and patient reported)

**Secondary outcomes:** Physical fitness, body composition, waist circumference, depression, physical activity level, arterial stiffness, endothelial function. Blood samples analyzed for both markers of disease activity and cardiovascular risk. Acute exercise responses: heart rate, respiration, oxygen uptake, plasma concentrations of lactate, interleukin-6, interleukin-18, tnf-alpha.

Contents

[Background 4](#__RefHeading___Toc295835609)

[Inflammatory markers 4](#__RefHeading___Toc295835610)

[Markers of cardiovascular disease and vascular function 4](#__RefHeading___Toc295835611)

[Exercise in patients with ankylosing spondylitis 5](#__RefHeading___Toc295835612)

[Acute responses to exercise in patients with ankylosing spondylitis 6](#__RefHeading___Toc295835613)

[Objective 6](#__RefHeading___Toc295835614)

[Methods 6](#__RefHeading___Toc295835615)

[Design 6](#__RefHeading___Toc295835616)

[Participants 7](#__RefHeading___Toc295835617)

[Sample size 7](#__RefHeading___Toc295835618)

[Randomization 7](#__RefHeading___Toc295835619)

[Assessments 8](#__RefHeading___Toc295835620)

[Outcome measures 8](#__RefHeading___Toc295835621)

[Exercise intervention 9](#__RefHeading___Toc295835622)

[Registration of adherence 10](#__RefHeading___Toc295835623)

[Statistical analyses 10](#__RefHeading___Toc295835624)

[Ethical considerations 10](#__RefHeading___Toc295835625)

[Time schedule 11](#__RefHeading___Toc295835626)

# Background

Ankylosing spondylitis (AS) is an inflammatory rheumatic disease affecting mainly the axial skeleton and sacroiliac joints presenting around 20-30 years of age (1). The overall prevalence is between 0.1 % and 1.4 % and higher among men than women, with a ratio of roughly 2 to 1 (1). Typical disease characteristics are inflammatory back pain, which may lead to structural and functional impairments, but AS may vary considerably with regard to disease symptoms and severity (2;3). Recently there has been an increased focus on the high risk of cardiovascular diseases (CVD) among patients with AS compared to the general population. The mortality rate among patients with AS is reported to be 1.7-1.9 higher than the general population, and the main reason for the increased mortality rate is cardiovascular events (4). The reason for the increased risk of cardiovascular diseases in patients with AS is still unclear. However, it is thought to be due to the inflammatory burden and an increased prevalence of traditional risk factors (5), which are factors that have an important role in accelerating atherosclerosis (6;7). There is a need for more research to explore the underlying mechanisms for the increased risk of CVD. In addition, interventions aiming at prevention of CVD should be investigated.

### Inflammatory markers in patients with AS

The exact level of inflammatory markers in patients with AS is not established, but studies have reported increased levels of C-reactive protein, sedimentation rate, tnf-alpha, interleukin 6, interleukin 18 (8-10). The recently launched AS disease activity score (ASDAS) is shown to reflect level of inflammation (11). This indicates that ASDAS can be used as an indirect measure of inflammatory status in patients with AS.

### Markers of cardiovascular disease and vascular function

There are several surrogate markers of vascular functions and atherosclerosis. Pulse wave velocity (PWV) analysis is a non-invasive way of assessing arterial stiffness, and a surrogate marker of vascular function. Increased arterial stiffness has been shown to correlate with coronary atherosclerosis and to predict coronary events and mortality in patients with hypertension (12). PWV is the gold standard of arterial stiffness measurements and has been reported to be reduced during anti-inflammatory treatment (13). Peripheral artery tonometry is another surrogate marker of cardiodiovascular disease and reflects endothelial function. It has been found to correlate strongly with coronary endothelial function and future cardiovascular events (14). In addition, reduced endothelial function is on of the first markers on atherosclerosis. The gold standard for measuring endothelial function is the flow mediated dilatation (FMD)-method and a reduction in FMD is often used as a early marker for arteriosclerosis (15). Studies have also showed that the developement og atherosklerosis include a chronich inflammatory prosess in the vascular wall and increased levels of vascular cell adhsions molecules (E-selectin, P-selectin and, ICAM-1 and VCAM-1) (16-18).

### Exercise as part of the disease management

Exercise is recommended as a cornerstone in the treatment of AS (19). A systematic review of exercise trials in patients with AS concluded that exercise has a beneficial effect on pain, spinal mobility, physical function and patient global assessment (20). The interventions in these trials focused mainly on flexibility training, and the effects on inflammatory markers and cardiovascular risk factors were not measured in any of the studies. However, in healthy adults’ cardiorespiratory exercise and muscular strength exercises are recommended for prevention of cardiovascular diseases (21). In line with this, intervention studies have shown that exercise have favorably effects on inflammation markers (22-24) and risk factors for cardiovascular diseases such as triglycerides(25), HDL-cholesterol(25), blood pressure(26), central obesity (27;28) and glucose tolerance(29) in healthy adults and other patients groups. Despite this, there is limited evidence on the effects of exercise on inflammation and traditional CV risk factors in patients with inflammatory rheumatic diseases. Intervention trials have shown that exercise has a favorable effect on body composition (30) and sedimentation rate (31) in patients with rheumatoid arthritis. Also in an observational study, Metsios et al. (32) found that physically inactive RA patients had a significantly higher systolic blood pressure, elevated total cholesterol and higher low-density lipoprotein cholesterol than physically active RA patients.

In line with this, in a recent cross sectional study, we found that cardiorespiratory fitness was inversely associated with disease activity in patients with AS. Furthermore, we found that high levels of cardiorespiratory fitness was significantly associated with lower risk of having metabolic syndrome, lower body mass index, smaller waist circumference, lower triglycerides and lower systemic inflammation in patients with AS and population controls (without rheumatic diseases). However, an important difference between population controls and patients was observed: high cardiorespiratory fitness was associated with lower blood pressure and higher levels of HDL-cholesterol in the controls, whereas these associations were absent in the patient group. Accordingly, there is a need to investigate the effects of exercise on inflammatory markers and cardiovascular risk factors in patients with AS.

### Acute responses to exercise in patients with AS

Even though exercise is recommended as part of the treatment, there is limited data on the acute physiological responses to exercise in AS patients. Carbon et al. examined responses to 30 min of cycling (33), and showed increased levels of neutrophils, leukocytes and lymphocytes which normalized quickly after exercise. The responses were similar to that seen in healthy controls. Interestingly, they also showed a small improvement in spinal flexibility immediately after exercise which normalized over the subsequent 3-5 hours. Exercise did also induce a marked increase in white blood cells, but there were no association between changes in the different variables. We have not been able to find any papers on how cytokines change with acute exercise. Of particular interest are the responses in inflammatory parameters such as interleukin-6, interleukin-18 and tnf-alpha. In addition, we want to examine cardiovascular and metabolic responses. Due to the underlying inflammation of AS patients, one might expect that they respond differently during an exercise bout. In addition, it will be of interest to investigate to what extent these responses are altered during long-term exercise.

# Objective

The aim of this study was twofold: firstly to investigate the effects of a supervised cardiorespiratory and muscle strengthening exercise program on disease activity and cardiovascular risk factors in patients with AS, and secondly, to examine acute effects on physiological responses to exercise.

# Methods

### Design

This study will be designed as a single blind randomized controlled trial.

### Participants

Patients who were classified with high disease activity in a cross sectional study carried out between 2008 and 2010 will be invited to participate (REK nr: s-03066 and S-02059). In addition, patients will be recruited from the rheumatologic outpatient clinic at Diakonhjemmet Hospital.

##### Inclusion criteria:

- Diagnosis of AS, confirmed by a rheumatologist
- Age, 18-70 -years.
- Not using TNF-α medication or steady medication for ≥3 months
- Disease activity ≥2.1 on ASDAS score defined as high disease activity (corresponding to a BASDAI score of ≥3.5).
- Not participated in a structured cardiorespiratory or muscle strengthening exercise program during the last year, including large amounts of brisk walking.

##### Exclusion criteria:

- Known cardiovascular disease.
- Severe comorbidity which involves reduced exercise capacity
- Not able to participate in weekly exercises sessions in Oslo.

### Sample size

To estimate the sample size we used the defined minimal clinical important difference in ASDAS score, which is 1.1 points (34). Based on a cross-sectional study recently carried out at our clinic we estimated the standard deviation to be 1.0. Power was set at 80% and α-value was set at 0.05 and drop-out rate was set at 20%. Based on these assumptions we estimated a sample size of 17 participants for each group. To ensure sufficient power also for secondary outcomes, a total of 40 participants are needed for this study.

### Randomization

A person, not involved in other aspects of the study, will assign the participants to either the exercise group (EG) or the control group (CG) following a computerised randomization program. A block randomization procedure will be used.

### Assessments

All assessments will be conducted at Diakonhjemmet Hospital. An independent assessor, unaware of the assignment to EG or CG, will examine the participants performed before randomization and after the intervention period.

### Outcome measures

##### Primary outcome: Disease activity

The AS Disease Activity Score-C-reactive protein (ASDAS-CRP) will be used to assess disease activity. ASDAS is reported to be associated with inflammatory biomarkers (11). The ASDAS is reported to have high face validity as it was developed through a Delphi process of experts and patients. It is a continuous measure based on patient-reported outcomes (back pain, duration of morning stiffness, patient global assessment and peripheral join complaints) and CRP, and higher values indicate higher disease activity. The minimal clinically important improvement for this instrument is reported to be ∆ ≥1.1, and ∆ ≥2.0 is considered a major improvement(34).

##### Secondary outcome measures

**Electrocardiography:** to measure the electrical activity of the heart.

**Blood samples:** Analyzed for both general and endothelial specific markers of inflammation and cardiovascular risk (NTproBNP, OPG, RANKL, VEGF, adhesion molecules, TNF-α, IL-6, IL-18, troponin T, high sensitive C-reactive protein, sedimentation rate, total cholesterol, LDL-cholesterol, HDL-cholesterol, triglycerides, apolipoprotein B, apolipoprotein A1, Lipoprotein A).

**Arterial stiffness and endothelial function:**  Measured using the Sphygmocor and Itamar respectively. In addition, the FMD-method will be used to measure endothelial function. Before examination of arterial stiffness at least four hours should have passed from the last meal, cigarette or cup of coffee. 10 hours should have passed from the last intake of alcohol.

**Blood pressure:** Resting diastolic-, systolic and mean arterial blood pressure will be measured.

**Cardiovascular health status:** The patients will be asked to complete a questionnaire reporting on the presence of CVD, the use of medication, smoking, and family history related to the incidence of CVD.

**Physical fitness:** Cardiorespiratory fitness will be assessed with an indirect maximal walking test on a treadmill for estimation of peak oxygen uptake according to modified Balke protocol. Hand grip strength will be assessed with GRIPPIT. Spinal and hip mobility will be assessed with the Bath Ankylosing Spondylitis Metrology index (BASMI), and chest expansion will be measured as the difference between maximal inspiration and expiration at the level of xipoideus (cm).

**Acute physiological responses to exercise:** Will be assessed with a submaximal exercise test (at a fixed work load) and as a part of the maximal treadmill test. The submaximal constant load test will be an integrated part of the warm-up procedure before the maximal treadmill test. Before and immediately after the submaxmial and the maximal bouts, blood samples will be taken. Additional samples will be taken during the maximal test and 5 min post exercise. The blood samples will be analyzed to determine the concentration of  interleukin 6 and 18, tnf-alpha and lactate. Heart rate will be recorded continually during and after the exercise bouts.

**Body composition:** Weight, height, waist circumference will be measured. Dual Energy X-ray Absortiometry (DEXA) will be used to assess body composition.

**Physical function:** Will be assessed with the patient reported index Bath Ankylosing Spondylitis Functional Index (BASFI).

**General health:** Will be assessed with the generic General Health Questionnaire (GHQ-12).

**Physical activity level:** Will be assessed with the International Physical Activity Questionnaire short version (IPAQ-s).

###

### Exercise intervention

The exercise intervention will be carried out at a fitness center with supervision from a physiotherapist.

A cardiorespiratory and muscle strengthening exercise program following the American College of Sports Medicine (ACSM) recommendations for maintenance and improvement of physical fitness. Cardiorespiratory fitness: two interval sessions (4 x 4 min), one continuous moderate exercise session (40 min) on a treadmill. The muscle strength exercises will consist of: 15-20 repetitions, large muscle groups as thighs, back and abdomen.

Dose: 12 weeks. Three times a week, 60 minutes.

Participants in the control group will be asked to continue their usual physical activity habits and will neither be encouraged nor discouraged from exercising. After the intervention period the control group will be offered an individually adapted exercise program and three exercise sessions with supervision from a physiotherapist.

### Registration of adherence

The participants will be asked to register exercise sessions in a diary and the physiotherapist will register attendance at the supervised exercise sessions. To fulfill the exercise protocol the participants must attend at least 80% of the planned exercise sessions.

Participants in the control group will be interviewed according their exercise habits between test 1 and 2.

# Statistical analyses

The main outcome measure will be analyzed as the between-group change in ASDAS score using ANVOVA with adjustments for baseline scores.

# Ethical considerations

All procedures will be carried out in accordance with the World Medical Association Declaration of Helsinki. The project will apply for approval by the National Committee for Medical Research Ethics, Southern Norway. The patients will be given oral and written information about the study and asked if they are willing to participate. In our opinion neither the testing nor the intervention causes any risk or disadvantages for the participants. Before the exercise intervention the participants will be examined by a Cardiologist to detect any contra-indication to exercise.

#

# Time schedule

**Autumn 2011:** Inclusions starts in week 33 and continues until 40 participants are included.

**Spring 2012:** The data collection will end within June 2012.

Reference List

(1) Braun J, Sieper J. Ankylosing spondylitis. Lancet 2007; 369(9570):1379-90.

(2) Spoorenberg A, van TA, Landewe R, Dougados M, van der Linden S, Mielants H et al. Measuring disease activity in ankylosing spondylitis: patient and physician have different perspectives. Rheumatology (Oxford) 2005; 44(6):789-95.

(3) Gran JT, Skomsvoll JF. The outcome of ankylosing spondylitis: a study of 100 patients. Br J Rheumatol 1997; 36(7):766-71.

(4) Peters MJ, van der Horst-Bruinsma IE, Dijkmans BA, Nurmohamed MT. Cardiovascular risk profile of patients with spondylarthropathies, particularly ankylosing spondylitis and psoriatic arthritis. Semin Arthritis Rheum 2004; 34(3):585-92.

(5) Peters MJ, Symmons DP, McCarey D, Dijkmans BA, Nicola P, Kvien TK et al. EULAR evidence-based recommendations for cardiovascular risk management in patients with rheumatoid arthritis and other forms of inflammatory arthritis. Ann Rheum Dis 2010; 69(2):325-31.

(6) Hannawi S, Haluska B, Marwick TH, Thomas R. Atherosclerotic disease is increased in recent-onset rheumatoid arthritis: a critical role for inflammation. Arthritis Res Ther 2007; 9(6):R116.

(7) Nagata-Sakurai M, Inaba M, Goto H, Kumeda Y, Furumitsu Y, Inui K et al. Inflammation and bone resorption as independent factors of accelerated arterial wall thickening in patients with rheumatoid arthritis. Arthritis Rheum 2003; 48(11):3061-7.

(8) Tutuncu ZN, Bilgie A, Kennedy LG, Calin A. Interleukin-6, acute phase reactants and clinical status in ankylosing spondylitis. Ann Rheum Dis 1994; 53(6):425-6.

(9) Park MC, Lee SW, Choi ST, Park YB, Lee SK. Serum leptin levels correlate with interleukin-6 levels and disease activity in patients with ankylosing spondylitis. Scand J Rheumatol 2007; 36(2):101-6.

(10) Bal A, Unlu E, Bahar G, Aydog E, Eksioglu E, Yorgancioglu R. Comparison of serum IL-1 beta, sIL-2R, IL-6, and TNF-alpha levels with disease activity parameters in ankylosing spondylitis. Clin Rheumatol 2007; 26(2):211-5.

(11) Pedersen SJ, Sorensen IJ, Garnero P, Johansen JS, Madsen OR, Tvede N et al. ASDAS, BASDAI and different treatment responses and their relation to biomarkers of inflammation, cartilage and bone turnover in patients with axial spondyloarthritis treated with TNF{alpha} inhibitors. Ann Rheum Dis 2011.

(12) Vlachopoulos C, Alexopoulos N, Stefanadis C. Aortic stiffness: prime time for integration into clinical practice? Hellenic J Cardiol 2010; 51(5):385-90.

(13) Angel K, Provan SA, Gulseth HL, Mowinckel P, Kvien TK, Atar D. Tumor necrosis factor-alpha antagonists improve aortic stiffness in patients with inflammatory arthropathies: a controlled study. Hypertension 2010; 55(2):333-8.

(14) Rubinshtein R, Kuvin JT, Soffler M, Lennon RJ, Lavi S, Nelson RE et al. Assessment of endothelial function by non-invasive peripheral arterial tonometry predicts late cardiovascular adverse events. Eur Heart J 2010; 31(9):1142-8.

(15) Toborek M, Kaiser S. Endothelial cell functions. Relationship to atherogenesis. Basic Res Cardiol 1999; 94(5):295-314.

(16) Morisaki N, Saito I, Tamura K, Tashiro J, Masuda M, Kanzaki T et al. New indices of ischemic heart disease and aging: studies on the serum levels of soluble intercellular adhesion molecule-1 (ICAM-1) and soluble vascular cell adhesion molecule-1 (VCAM-1) in patients with hypercholesterolemia and ischemic heart disease. Atherosclerosis 1997; 131(1):43-8.

(17) Ridker PM, Hennekens CH, Roitman-Johnson B, Stampfer MJ, Allen J. Plasma concentration of soluble intercellular adhesion molecule 1 and risks of future myocardial infarction in apparently healthy men. Lancet 1998; 351(9096):88-92.

(18) Schumacher A, Seljeflot I, Sommervoll L, Christensen B, Otterstad JE, Arnesen H. Increased levels of markers of vascular inflammation in patients with coronary heart disease. Scand J Clin Lab Invest 2002; 62(1):59-68.

(19) Zochling J, van der HD, Burgos-Vargas R, Collantes E, Davis JC, Jr., Dijkmans B et al. ASAS/EULAR recommendations for the management of ankylosing spondylitis. Ann Rheum Dis 2006; 65(4):442-52.

(20) Dagfinrud H, Kvien TK, Hagen KB. Physiotherapy interventions for ankylosing spondylitis. Cochrane Database Syst Rev 2008;(1):CD002822.

(21) Haskell WL, Lee IM, Pate RR, Powell KE, Blair SN, Franklin BA et al. Physical activity and public health: updated recommendation for adults from the American College of Sports Medicine and the American Heart Association. Med Sci Sports Exerc 2007; 39(8):1423-34.

(22) Troseid M, Lappegard KT, Mollnes TE, Arnesen H, Seljeflot I. The effect of exercise on serum levels of interleukin-18 and components of the metabolic syndrome. Metab Syndr Relat Disord 2009; 7(6):579-84.

(23) Bruunsgaard H. Physical activity and modulation of systemic low-level inflammation. J Leukoc Biol 2005; 78(4):819-35.

(24) Troseid M, Lappegard KT, Claudi T, Damas JK, Morkrid L, Brendberg R et al. Exercise reduces plasma levels of the chemokines MCP-1 and IL-8 in subjects with the metabolic syndrome. Eur Heart J 2004; 25(4):349-55.

(25) Durstine JL, Grandjean PW, Cox CA, Thompson PD. Lipids, lipoproteins, and exercise. J Cardiopulm Rehabil 2002; 22(6):385-98.

(26) Fagard RH, Cornelissen VA. Effect of exercise on blood pressure control in hypertensive patients. Eur J Cardiovasc Prev Rehabil 2007; 14(1):12-7.

(27) Irwin ML, Yasui Y, Ulrich CM, Bowen D, Rudolph RE, Schwartz RS et al. Effect of exercise on total and intra-abdominal body fat in postmenopausal women: a randomized controlled trial. JAMA 2003; 289(3):323-30.

(28) Hellenius ML, de FU, Berglund B, Hamsten A, Krakau I. Diet and exercise are equally effective in reducing risk for cardiovascular disease. Results of a randomized controlled study in men with slightly to moderately raised cardiovascular risk factors. Atherosclerosis 1993; 103(1):81-91.

(29) Hellenius ML, Brismar KE, Berglund BH, de Faire UH. Effects on glucose tolerance, insulin secretion, insulin-like growth factor 1 and its binding protein, IGFBP-1, in a randomized controlled diet and exercise study in healthy, middle-aged men. J Intern Med 1995; 238(2):121-30.

(30) Lemmey AB, Marcora SM, Chester K, Wilson S, Casanova F, Maddison PJ. Effects of high-intensity resistance training in patients with rheumatoid arthritis: a randomized controlled trial. Arthritis Rheum 2009; 61(12):1726-34.

(31) Hakkinen A, Hannonen P, Nyman K, Lyyski T, Hakkinen K. Effects of concurrent strength and endurance training in women with early or longstanding rheumatoid arthritis: comparison with healthy subjects. Arthritis Rheum 2003; 49(6):789-97.

(32) Metsios GS, Stavropoulos-Kalinoglou A, Panoulas VF, Wilson M, Nevill AM, Koutedakis Y et al. Association of physical inactivity with increased cardiovascular risk in patients with rheumatoid arthritis. Eur J Cardiovasc Prev Rehabil 2009; 16(2):188-94.

(33) Carbon RJ, Macey MG, McCarthy DA, Pereira FP, Perry JD, Wade AJ. The effect of 30 min cycle ergometry on ankylosing spondylitis. Br J Rheumatol 1996; 35(2):167-77.

(34) Machado P, Landewe R, Lie E, Kvien TK, Braun J, Baker D et al. Ankylosing Spondylitis Disease Activity Score (ASDAS): defining cut-off values for disease activity states and improvement scores. Ann Rheum Dis 2010; [doi]10.1136/ard.2010.138594.
